# Current Status of Marine-Derived Compounds as Warheads in Anti-Tumor Drug Candidates

**DOI:** 10.3390/md15040099

**Published:** 2017-03-29

**Authors:** David J. Newman, Gordon M. Cragg

**Affiliations:** 1 Wayne, PA 19087, USA; 2 Gaithersburg, MD 20877, USA; gmcragg2@gmail.com

**Keywords:** Antibody Drug Conjugates (ADCs), marine derived antitumor agents, clinical trials

## Abstract

In this review, we have attempted to describe all of the antibody–drug conjugates using a marine-derived compound as the “warhead”, that are currently in clinical trials as listed in the current version of the NIH clinical trials database (clinicaltrials.gov). In searching this database, we used the beta-test version currently available, as it permitted more specific search parameters, since the regular version did not always find trials that had been completed in the past with some agents. We also added small discussion sections on candidates that are still at the preclinical stage, including a derivative of diazonamide that has an unusual interaction with tubulin (DZ-23840), which may also be a potential warhead in the future.

## 1. Introduction

In early 2014, Newman and Cragg reviewed the then status of antitumor and pain control agents whose base structures were related to or sourced from marine sources [1], which was followed by a more generalized short review requested for a Special Issue of Planta Medica published early in 2016 [2].

Since the field is moving rapidly, with significant numbers of monoclonal antibodies (mAbs) directed against disparate targets being reported regularly in the scientific and clinical literature, a potential “ADC compound” (antibody drug conjugate) may be in clinical trials one year and then not be “in play” the next year, or in some cases within 6–9 months of an initial report, or may never actually reach a patient for one reason or another.

The ADCs that we are reporting in this short review are those that, with some exceptions, have reached clinical trials and where at all possible, we will list the relevant trial numbers in the NIH Clinical Trial database.

What we have decided to do is to report the ADCs sorted by their “warhead” without taking into account, the class of linker between the “warhead” and the mAb. In addition, if there are subtle changes in the warhead from a known structure, then we will discuss these and show what may be a candidate for other mAbs in the future. We realize that this is not a “complete” account, as at times, reports are made in the literature using only a code name for an ADC and no information as to the “warhead”. Thus, this review is as complete as we can make it using publicly available resources, but it will not contain every marine-derived warhead extant.

## 2. Dolastatin 10 and Related Structures

The isolation and identification by the Pettit group of dolastatin 10 was reported in 1987 [3]. Due to the very low levels of naturally occurring dolastatins, Pettit (the original discoverer of this highly potent series) and collaborators were forced to develop novel synthetic methods in order to obtain enough material to perform even basic cell biology, let alone perform/provide material for further development. The initial publication led to a series of others describing the synthetic processes. Rather than list the significant number of papers covering these details, the current status as of 1997, giving details of this agent and “relatives”, their activities and methods of synthesis are recorded in an excellent review by Pettit [4]. A further review by Flahive and Srirangam (with additions by the editors of the book), brought the story up to 2011 [5].

**Figure 1:**
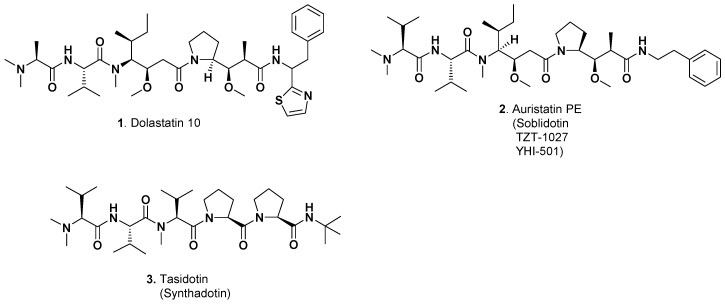
Single Dolastatin-derived Agents in Anticancer Trials (Structures **1**–**3**).

**Figure 2:**
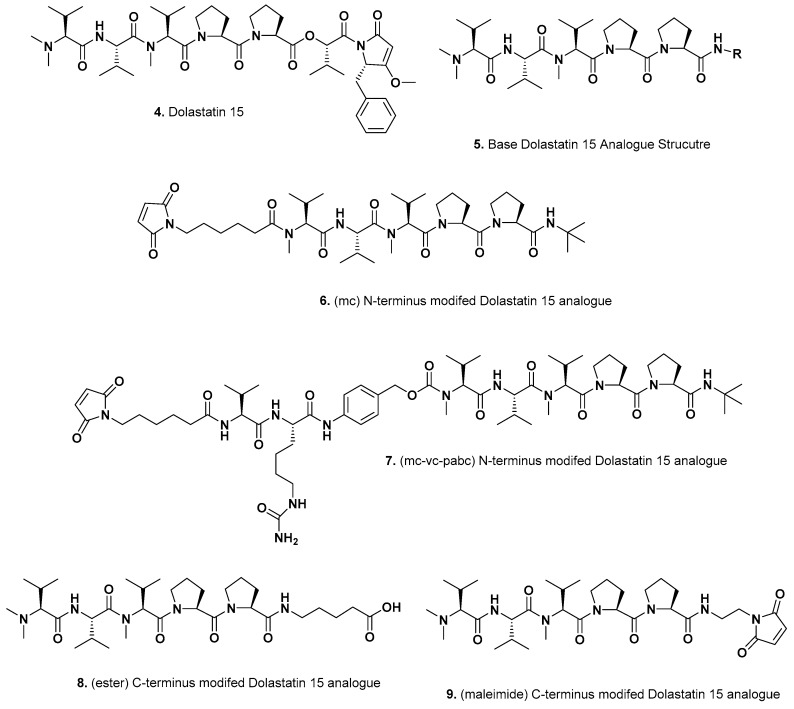
Dolastatin 15 Derivatives as ADC Warheads by Genzyme (Structures **4**–**9**).

**Figure 3:**
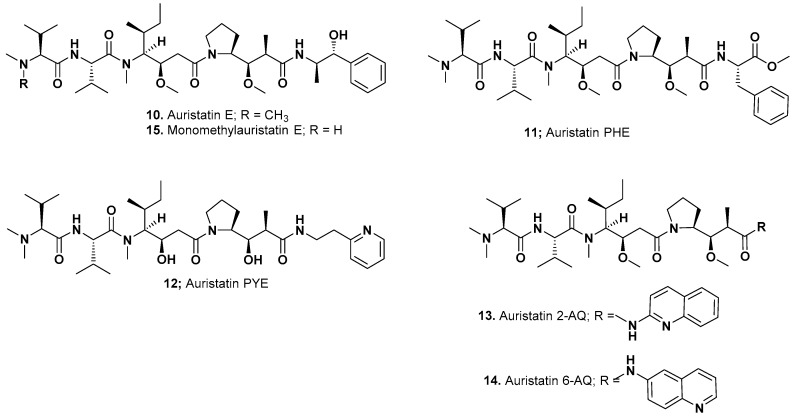
Auristatin Derivatives from the Pettit Group (Structures **10**–**15**).

As mentioned above, due to the extremely potent cytotoxic activities displayed by dolastatin 10 (Figure 1; **1**) which was identified as a tubulin interactive agent, the Pettit group and others synthesized variations on the structure of dolastatin 10. In addition to the papers referred to above, further details of the extensive studies leading to identification of compounds with significant activities can be found in both the scientific literature [6,7], and also in the patent literature [8]. Related derivatives, auristatin PE (Figure 1; **2**) (also known as soblidotin, TZT-1027, and YHI-501), and tasidotin (Figure 1; **3**) entered clinical trials, though none proceeded beyond Phase II, due to toxicity and/or a lack of efficacy in the trials. Using the NIH Clinical Trials database and looking for all trials of these three agents, the following results were found (as of 25FEB17) with all trials in the late 1990s or early 2000s as start dates: dolastatin 10 with seven trials showing completed with details published on a Phase I trial with modest activity [9]; a Phase II trial in prostate cancer that had no efficacy [10]; and a Phase II trial against advanced breast cancer that, quoting from the authors, “it had minimal activity in this advanced breast cancer study. We are not pursuing further clinical trials of this agent in the setting of advanced breast cancer” [11]. This was the last recorded data from a clinical trial of dolastatin 10 as a single agent. With the other derivatives, there was a Phase II trial of auristatin PE (as soblidotin), two trials at Phase II, and one at Phase I that was terminated before starting. Tasidotin as ILX651 had three trials, all in the early 2000s, with no reports at the Phase II level. With no significant results published in the last 10 years, it is safe to assume that these agents are no longer being used as single or even components of multiple agents today.

## 3. Dolastatin 15 and Variants as Warheads

**Figure 4:**
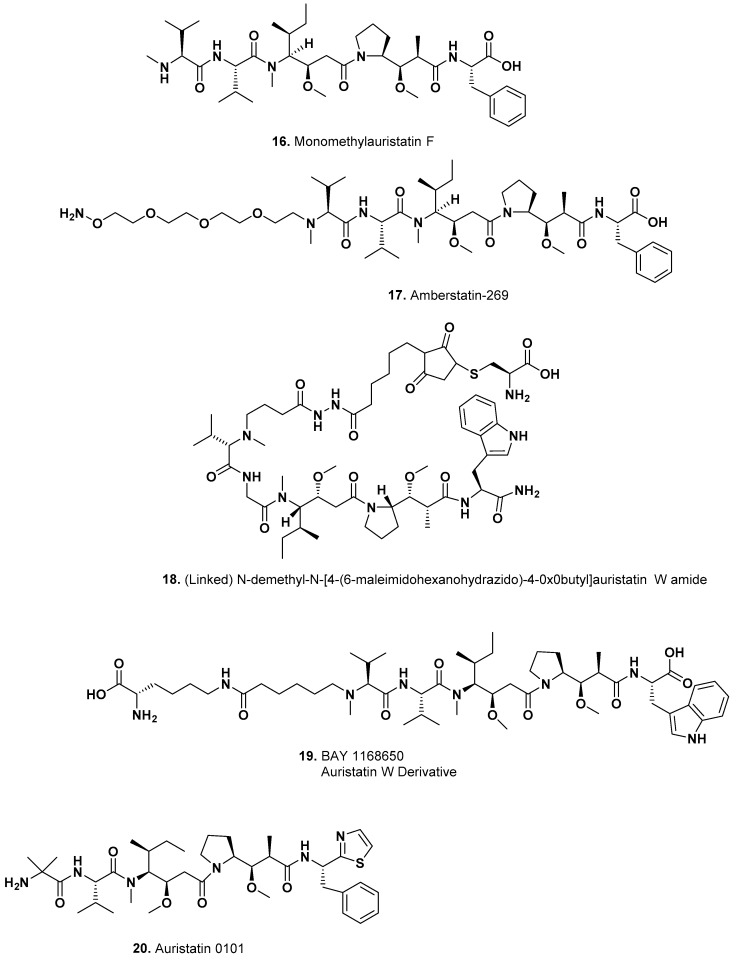
MMAF and Other Modified Auristatin Molecules in Clinical Trials (Structures **16**–**20**).

**Figure 5:**
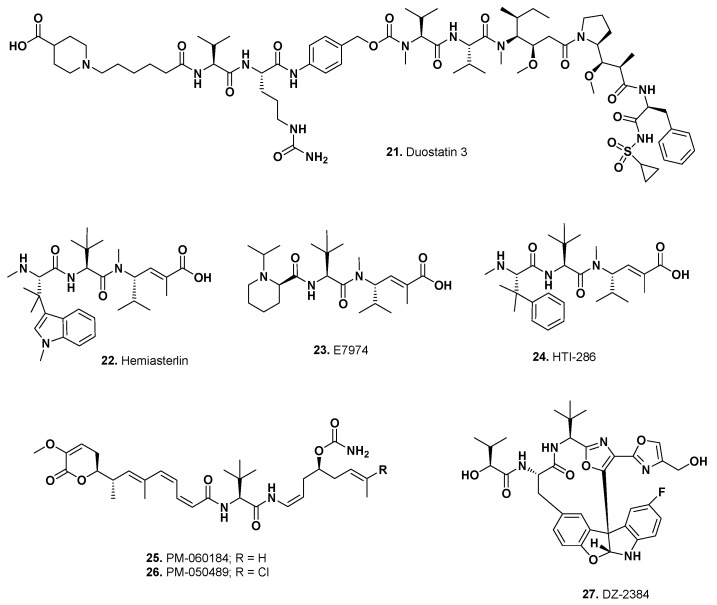
Preclinical Candidates; Auristatin and Other Structure-Based Molecules (Structures **21**–**27**).

In 2012, a group at Genzyme [12] reported that they had utilized another linear dolastatin peptide, dolastatin 15 (Figure 2; **4**), as a potential warhead, and reported on four variations designed to give different linkage sites at their *N* and *C* termini. Modifying the dolastatin 15 base structure by removing the last two residues on the *C*-terminal end gave structure (Figure 2; **5**) which followed from the report by Pettit et al. in 1998 [13], where they demonstrated that replacement of the *C*-terminal (*S*)-dolapyrrolidinone unit by a number of diverse amides still maintained the anti-tubulin activities. To these base structures the Genzyme scientists linked their *N* termini to traztuzumab via a maleimidocaproyl (MC); (Figure 2; **6**) or a maleimido-caproyl-valine-citrulline-*p*-aminobenzyloxy-carbonyl linker (MC-VC-PABC); (Figure 2; **7**) using partially reduced interchain disulfides on the traztuzumab molecule. With the *C*-termini, a conjugate based on esterification using structure (Figure 2; **8**) or a maleimide entity (Figure 2; **9**) were used with all structures shown in Figure 2. Although these agents when linked at the *C*-terminus demonstrated activity against her2neu-expressing cells, the *N*-terminal-linked ADC did not. The ester-linked variant (Figure 2; **8**) did demonstrate excellent in vivo activity in SCID mice but no further work has been reported with these agents. However, the work did demonstrate that truncated dolastatin structures do have potential.

The use of traztuzumab was probably based upon the success of the maytansine-linked mAb (Kadcyla^®^) that was approved in the same time frame by the FDA, coupled to the linkers from the data from the Senter group at Seattle Genetics using auristatins as warheads.

## 4. Auristatins

### 4.1. Background

As mentioned earlier, in 1995 Pettit reported the syntheses of a variety of peptides related to dolastatin 10 [6,8] and followed these initial reports in 1998 with further studies on dolastatin 10 SAR probes [7]. Then, in 2011, his group reported modifications [14] with increased water solubility, whilst still maintaining significant potency against tumor lines (10–100 pM depending upon the lines used). The syntheses and structures of other modifications are given at length in the papers referred to above [6,7,8,14]. These structures, included auristatin E (Figure 3; **10**), auristatin PHE (Figure 3; **11**), auristatin PYE (Figure 3; **12**), and two interesting aminoquinoline derivatives, auristatin-2-AQ (Figure 3; **12**) and auristatin-6-AQ (Figure 3; **14**).

### 4.2. Antibody Drug Conjugates (General Patent Status)

A very interesting recent paper that is well worth reading for scientists who are interested in the overall aspects of antibody conjugates, is a 2017 one covering the intellectual property considerations of these agents [15]. The various linkers and their patent status are discussed at length by the author.

### 4.3. Approved Monomethylauristatin-Linked ADCs (MMAE-ADC)

In 2003, Seattle Genetics scientists published an excellent paper on the early development of mAbs linked to both auristatin E (Figure 3; **10**) or a new analogue, monomethyl auristatin E (or MMAE; Figure 3; **15**) with a base patent published in 2002 [16]. Seattle Genetics used either acid labile hydrazine linkers or a valine-citrulline or a phenyl-lysine linked to MMAE, since the last two conjugation systems were more stable than the hydrazone of 5-benzoylvaleric acid-auristatin E ester (AEVB) linked at the *C*-terminus [17]. A recent discussion on the site dependency of non-cleavable auristatin derivatives (relevant to MMAF conjugates) should be read as it covers the reasons for specific methodologies [18], together with another from the same group covering cleavable sites [19]. In addition, it is also well worth consulting a slightly earlier paper on this technology [20].

These initial series of experiments led to the identification, clinical trials and FDA approval of the first mAb-linked auristatin derivative, brentuximab vedotin (Adcetris^®^) in 2011, with an excellent report on its developmental history being published by Senter and Sievers of Seattle Genetics in 2012 [21]. Initially, this ADC had the chimeric anti-CD30 antibody cAC10 (SGN-30), linked through their valine-citrulline linker to an average of eight molecules of MMAE. Subsequent development work reduced the loading to four molecules of MMAE [21]. Seattle Genetics entered into a co-development program with Millennium Pharmaceuticals for lymphoma and anaplastic large cell lymphoma, with Seattle Genetics having rights in the US/Canada and Millenium/Takeda having rights in the EU. The ADC has also been approved in Japan under the auspices of Takeda (who own Millenium).

Currently, this ADC, as is customary with most approved anti-tumor treatments, is in further clinical trials (Phases I–III) against multiple types of lymphoma and leukemia in the US and in other countries.

There are currently 88 entries from Phase I to Phase III listed in the clinical trials database as of the middle of March 2017, when searching for each trial phase. Due to the numbers involved, we have given very brief details for each stage below.

- Phase III: seven (six being directed against various lymphomas with multiple drug regimens, and one being a comparative trial).
- Phase II: 53 (52 against various lymphomas with 15 only studying brentuximab vedotin, 35 having a variety of other drug treatments in addition to the ADC, and one where the ADC alone is being tested against mesothelioma).
- Phase I: 28 (27 against lymphomas and leukemias, with one against mycosis fungoides/Sezary Syndrome. One trial has brentuximab vedotin as the sole agent studying Graft versus Host Disease following allogenic stem cell transplantation).

That this ADC may be used in non-adult patients was shown by the recent interesting report demonstrating that it may be used in pediatric patients, with the major limitation with respect to toxicity concerns being the weight of the patient [22].

### 4.4. Monomethylauristatin E-Linked ADCs in Clinical Trials

At the time of writing this review (late February 2017), the following MMAE-linked ADCs are in clinical trials, but we should point out that in a few cases we have reported agents that have been in clinical trials, but whose current status is not too clear. ADCs will be listed not in alphabetical or calendar order but by level of trial, starting with Phase II, though it must be made clear that compounds may be in different trial phases at the same time. If so then it will be noted under the highest phase. Where there are multiple trials under a given ADC name, we have tried to list them in descending numerical order, though this may not always be the case, particularly if in a single Phase I that may develop into a Phase II trial. The short title in the NIH database is given after the NCT number for reference, and where the ADC is in different level trials under the same NCT number, this is noted.

#### 4.4.1. Glembatumumab Vedotin (Phase II)

This ADC is monomethyl auristatin E (MMAE) linked to the anti-CG56972 fully human monoclonal antibody CR011, through the valine-citrulline dipeptide linker under the auspices of Celldex (originally named CuraGen). The antibody component is the fully human mAb (CR-011) generated by a subsidiary of Amgen. Though not a clinical trial, a recent publication demonstrated that this ADC may have utility in pediatric sarcoma patients, and a clinical trial to study this has been suggested [23].

Currently the following trials are underway/recruiting. In this particular case, we have not listed completed trials.

- NCT02713828: Glembatumumab Vedotin in gpNMB-Expressing, Advanced or Metastatic SCC of the Lung (PrE0504), Phases I/II.
- NCT02487979: Glembatumumab Vedotin in Treating Patients with Recurrent or Refractory Osteosarcoma, Phase II (pharmacokinetics as well).
- NCT02363283: Glembatumumab Vedotin in Treating Patients with Metastatic or Locally Recurrent Uveal Melanoma, Phase II.
- NCT02302339: A Study of Glembatumumab Vedotin as Monotherapy or in Combination with Immunotherapies in Patients with Advanced Melanoma, Phase II.
- NCT01997333: Study of Glembatumumab Vedotin (CDX-011) in Patients with Metastatic, gpNMB Over-Expressing, Triple Negative Breast Cancer (METRIC), Phase II.

#### 4.4.2. Pinatuzumab Vedotin (RG-7593) (Phases I/II)

This ADC is monomethylauristatin E (MMAE) conjugated via the usual valine-citrulline linker to the humanized IgG1-kappa anti-CD22 monoclonal antibody, with studies under the auspices of Genentech.

- NCT01691898: A Study of Pinatuzumab Vedotin (DCDT2980S) Combined with Rituximab or Polatuzumab Vedotin (DCDS4501A) Combined with Rituximab or Obinutuzumab in Participants with Relapsed or Refractory B-Cell Non-Hodgkin’s Lymphoma (ROMULUS), Phases I/II.

#### 4.4.3. Polatuzumab Vedotin (RG-7596) (Phases I/II)

This ADC is an anti-CD79 humanized monoclonal antibody conjugated to monomethylauristatin E (MMAE) via the usual linker (mc-valine-citrulline-PABC), and is being developed by Genentech and Chugai depending upon the specific geographic area.

- NCT01992653: A Study of Polatuzumab Vedotin in Combination with Rituximab or Obinutuzumab, Cyclophosphamide, Doxorubicin, and Prednisone in Participants with B-Cell Non-Hodgkin’s Lymphoma, Phases I/II.
- NCT01691898: A Study of Pinatuzumab Vedotin (DCDT2980S) Combined with Rituximab or Polatuzumab Vedotin (DCDS4501A) Combined with Rituximab or Obinutuzumab in Participants with Relapsed or Refractory B-Cell Non-Hodgkin’s Lymphoma (ROMULUS), Phases I/II. (Note that this trial also includes pinatuzumab vedotin).
- NCT02729896: A Study of Obinutuzumab, Polatuzumab Vedotin, and Atezolizumab in Relapsed or Refractory Follicular Lymphoma (FL) or Diffuse Large B-Cell Lymphoma (DLBCL), Phase I (under Hofmann-LaRoche).
- NCT02611323: A Study of Obinutuzumab, Polatuzumab Vedotin, and Venetoclax in Relapsed or Refractory Follicular Lymphoma (FL) or Diffuse Large B-Cell Lymphoma (DLBCL). Phase I (under Hofmann-LaRoche).
- NCT02600897: A Study of Obinutuzumab, Polatuzumab Vedotin, and Lenalidomide in Relapsed or Refractory Follicular Lymphoma (FL) or Diffuse Large B-Cell Lymphoma (DLBCL). Phase I (under Hofmann-LaRoche).
- NCT02257567: A Study of Polatuzumab Vedotin (DCDS4501A) in Combination with Rituximab or Obinutuzumab Plus Bendamustine in Participants with Relapsed or Refractory Follicular or Diffuse Large B-Cell Lymphoma. Phase I/II (under Hofmann-LaRoche).

#### 4.4.4. Lifastuzumab Vedotin (RG-7599; Also Known as DNIB-0600A) (Phases I/II)

This ADC is a humanized IgG1 mAb that targets the “anti-sodium-dependent Phosphate Transport Protein 2B” (SLC34A2 or NaPi-2b). This is under the auspices of Genentech.

- NCT01991210: A Study of DNIB0600A in Comparison with Pegylated Liposomal Doxorubicin (PLD) in Participants with Platinum-Resistant Ovarian Cancer (PROC). Phase II.
- NCT01995188: A Study to Evaluate the Safety and Pharmacology of DNIB0600A in Participants with Platinum-Sensitive Ovarian Cancer or Non-Squamous Non-Small Cell Lung Cancer. Phase I.
- NCT01363947: Safety and Pharmacokinetics of Escalating Doses of DNIB0600A in Patients with Non-Small Cell Lung Cancer and Platinum Resistant Ovarian Cancer. Phase I.

#### 4.4.5. Tisotumab Vedotin (Phases I/II)

This ADC is a tissue factor-specific human IgG1k antibody (TF-011) conjugated to monomethylauristatin E (MMAE) via the usual valine-citrulline linker and is under active clinical trials by Genmab. It is designed for use by patients who have failed regular treatments. Three recent papers discuss this construct, which was originally known as “HuMax-TF-ADC”, and should be consulted for the reasoning behind the choices made [24,25,26].

- NCT02552121: Tisotumab Vedotin (HuMax^®^-TF-ADC) Safety Study in Patients with Solid Tumors. This is a dose-escalating and cohort expansion study. Phase I/II.
- NCT02001623: Tisotumab Vedotin (HuMax^®^-TF-ADC) Safety Study in Patients with Solid Tumors. This is the first in human trial. Phase I/II.

#### 4.4.6. PSMA-ADC (Phases I/II)

This ADC is a human dimeric-specific PSMA monoclonal antibody conjugated to vcMMAE under the auspices of Progenics. Almost all publications on this ADC are in the form of abstracts, but in 2011, a full paper was published on in vitro/in vivo experiments by Wang et al. [27]. Currently there are five completed trials reported in the NCT database and the ADC appears to still be under development. Toxicities have been recently reported by Donaghy [28].

- NCT02020135: An Open-label Treatment Extension of PSMA ADC in Subjects with Metastatic Castration-resistant Prostate Cancer (mCRPC). Phase II: Study completed.
- NCT01856933: BrUOG 263: Prostate Specific Membrane Antigen (PSMA) Glioblastoma Multiforme (GBM). Phase II: Study completed.
- NCT01695044: A Study of PSMA ADC in Subjects with Metastatic Castration-resistant Prostate Cancer (mCRPC). Phase II: Study completed.
- NCT01414296: Prostate-specific Membrane Antigen Antibody-Drug Conjugate in Subjects with Prostate Cancer. Phase I: Study completed.
- NCT01414283: Prostate-specific Membrane Antigen Antibody-Drug Conjugate in Subjects with Prostate Cancer. Phase I: Study completed.

#### 4.4.7. MLN-0264 (Industuzumab Vedotin) (Phases I/II)

This ADC is a human mAb (SF9) linked to vcMMAE and targeted against guanyl cyclase C. It is still in clinical trials from the final collection dates reported in some of the clinical trials but appears that it might have been discontinued from further development. Details as to its earlier status are given by Thomas et al. [29] and toxicities by Donaghy [28].

- NCT02391038: MLN0264 in Previously Treated Asian Patients with Advanced Gastrointestinal Carcinoma or Metastatic or Recurrent Gastric or Gastroesophageal Junction Adenocarcinoma Expressing Guanylyl Cyclase C. Phase I/II: Completed.
- NCT02202785: A Study of MLN0264 in Patients with Pancreatic Cancer. Continuing but not recruiting. Phase II: Final date for data collection is May 2018.
- NCT02202759: A Study of MLN0264 in Patients with Cancer of the Stomach or Gastroesophageal Junction. Continuing but not recruiting. Phase II: Final date for data collection is September 2018.
- NCT01577758: Study of MLN0264 in Adult Patients with Advanced Gastrointestinal Malignancies Expressing Guanylyl Cyclase C. Phase I: The study was completed in February 2014.

#### 4.4.8. DEDN-6256A (RG-7636) (Phase I)

This is a Genentech ADC that uses an Endothelin ETB receptor as the target of the mAb linked to MMAE. No details as to the mAb or the linker are given. Toxicity results were reported by Donaghy [28], and initial results in abstract form in 2014 by Infante et al. [30].

- NCT01522664: A Study of DEDN6526A in Patients with Metastatic or Unresectable Melanoma. Phase I: The study is completed.

#### 4.4.9. DMOT-4039A (Phase I)

This is a Genentech ADC that is the mAb MMOT-0530A, targeting an antigen overexpressed in pancreatic and ovarian cancer cells, conjugated to MMAE but with no further details as to the nature of the linkage. Details are given in the papers by Zhao et al. [31], Hassan et al. [32], and Lamberts et al. [33], with toxicity data from Donaghy [28], and with initial clinical reports published by Weekes et al. [34].

- NCT01832116: 89Zr-MMOT PET Imaging in Pancreatic and Ovarian Cancer Patients (MMOT). Phase I: Completed with data published [33].
- NCT01469793: A Study of DMOT4039A in Participants with Unresectable Pancreatic or Platinum-Resistant Ovarian Cancer. Phase I: Completed with a paper covering the results published in 2016 [34].

#### 4.4.10. Enfortumab Vedotin (Phase I)

This ADC is the human anti-nectin-4 antibody conjugated to MMAE via the customary valine-citrulline linker. Trials are underway/completed at the Phase I level in patients with metastatic urothelial cancer that expresses Nectin-4. The validity of the treatment was shown in a recent publication at the preclinical level by Challita-Eid et al. [35]. The clinical sponsor is Agensys (Astellas Pharma) and the NCT data-tables use ASG-22CE as the search parameter.

- NCT02091999: A Study of Escalating Doses of ASG-22CE Given as Monotherapy in Subjects with Metastatic Urothelial Cancer and Other Malignant Solid Tumors That Express Nectin-4. Phase I.
- NCT01409135: A Study of the Safety and Pharmacokinetics of ASG-22M6E in Subjects with Malignant Solid Tumors That Express Nectin-4. Phase I.

#### 4.4.11. Telisotuzumab Vedotin (Phase I)

This ADC is a humanized monoclonal IgG1 kappa, anti-human MET receptor (plus other related targets) linked via the customary valine-citrulline-MMAE warhead. It is sponsored by AbbVie and, as with other agents, searches have to use ABBV-399 in the NCT data-tables. A report of early results from the Phase I study suggesting efficacy was published by Wang et al. in 2017 [36].

- NCT02099058: A Phase I/Ib Study with ABBV-399, an Antibody Drug Conjugate, in Subjects with Advanced Solid Cancer Tumors. Phase I.

#### 4.4.12. DLYE5953A (Phase I)

This ADC is a humanized IgG1 monoclonal antibody that targets the lymphocyte antigen 6 complex (LY6E) conjugated to MMAE, under the auspices of Genentech. The linker is not given but is probably based on valine-citrulline.

- NCT02092792: A Study Evaluating the Safety of Escalating Doses of DLYE5953A in Patients with Refractory Solid Tumors. Phase I: This trial is still continuing but not recruiting after November 2016.

#### 4.4.13. SGN-LIV1A (Phase I)

This ADC is a Seattle Genetics construct of an anti-LIV-1 monoclonal antibody linked to MMAE. This is designed for treatment against metastatic breast cancer targeting the zinc transporter LIV-1 (SLC39A6) with further details in the report by Sussman et al. [37].

- NCT01969643: A Safety Study of SGN-LIV1A in Breast Cancer Patients. Phase I.

#### 4.4.14. ASG-15E/15ME (Phase I)

This ADC is a fully human IgG2 monoclonal antibody (AGS15) targeting SLITRK6 conjugated to valine-citrulline-MMAE under the auspices of Astellas Pharma. Details on its development are given in the recent paper by Morrison et al. [38].

- NCT01963052: ASG-15ME is a Study of Escalating Doses of AGS15E Given as Monotherapy in Subjects with Metastatic Urothelial Cancer. Phase I.

#### 4.4.15. AGS-67E (Phase I)

This ADC is a human mAb directed against CD37conjugated to MMAE and is under the auspices of Astellas Pharma and Seattle Genetics. A paper covering some of the early results was published in 2015, which should be consulted for details as to why the target was chosen [39].

- NCT02610062: A Study to Evaluate Safety, Tolerability, and Pharmacokinetics of Escalating Doses of AGS67E Given as Monotherapy in Subjects with Acute Myeloid Leukemia (AML). Phase I: This trial is actively recruiting.
- NCT02175433: A Study to Evaluate Safety, Tolerability, and Pharmacokinetics of Escalating Doses of AGS67E Given as Monotherapy in Subjects with Refractory or Relapsed Lymphoid Malignancies. Phase I: This trial is actively recruiting.

#### 4.4.16. ASG-5ME (Phase I)

This ADC is targeted against the solute carrier receptor SLC44A4, and is a human IgG2 ant-SLC44A4 mAb linked to valine-citrulline-MMAE. It entered clinical trials at Phase I under Seattle Genetics but was discontinued for “commercial reasons”, with no further details being published. The work leading up to its discovery and preclinical development was published in 2016 by Mattie et al. [40], and from the details reported by Covaler et al. in 2016 on the 01166490 trial, a potential reason for stopping development was the low response level seen [41].

- NCT01228760: A Study to Determine the Maximum Tolerated Dose of ASG-5ME in Subjects with Castration-Resistant Prostate Cancer. Phase I: Completed.
- NCT01166490: Dose Escalation Trial of ASG-5ME in Pancreatic or Gastric Adenocarcinoma. Phase I: Completed.

#### 4.4.17. DMUC-5754A (Sofituzumab Vedotin) (Phase I)

This is a Genentech construct of a mAb against MUC16 linked to MMAE, probably via the valine-citrulline linker. It was in a Phase I trial where positive responses were seen in patients with high MUC16 levels [42], but no developments seem to have occurred since the middle of 2015.

- NCT01335958: Safety and Pharmacokinetics of DMUC5754A Administered Intravenously to Patients with Platinum-Resistant Ovarian Cancer or Unresectable Pancreatic Cancer. Phase I: Completed and details published [42].

#### 4.4.18. RG7450 (Vandortuzumab Vedotin) (Phase I)

This is an ADC consisting of a humanized anti-STEAP1 IgG1 antibody which is a thio-anti-STEAP1 (ThioMab) variant, coupled to MMAE through the linker MC-valine-citrulline-PAB [43]. It was placed into a Phase I clinical trial under the auspices of Genentech under the code name DSTP3086S.

- NCT01283373: A Study of the Safety and Pharmacokinetics of Escalating Doses of DSTP3086S in Patients with Metastatic Castration-Resistant Prostate Cancer. Phase I: This trial was completed but no information on further work has been available since early 2016.

#### 4.4.19. DFRF4539A (Phase I)

This ADC is composed of a monoclonal antibody directed against a specific myeloma antigen and conjugated to MMAE. It entered a Phase I trial under Genentech in 2011 and was completed in 2014. No details have been published yet.

- NCT01432353: A Study of DFRF4539A in Patients with Relapsed or Refractory Multiple Myeloma. Phase I

### 4.5. Monomethylauristatin F-Linked ADCs in Clinical Trials

#### 4.5.1. Denintuzumab Mafodotin (SGN-CD19A) (Phases I/II)

This ADC is a humanized antibody targeting CD19 conjugated to monomethylauristatin F (MMAF; Figure 4; **16**) through a maleimidocaproyl (mc) linker [29].

- NCT02855359: Denintuzumab Mafodotin (SGN-CD19A) Combined with RCHOP or RCHP versus RCHOP Alone in Diffuse Large B-Cell Lymphoma or Follicular Lymphoma. Phase II (recruiting).
- NCT02592876: Treatment Study of Denintuzumab Mafodotin (SGN-CD19A) Plus RICE Versus RICE Alone for Diffuse Large B-Cell Lymphoma. Phase II (recruiting).
- NCT01786135: A Safety Study of SGN-CD19A for B-Cell Lymphoma. Phase I: Study ongoing but not recruiting.
- NCT01786096: A Safety Study of SGN-CD19A for Leukemia and Lymphoma. Phase I: Study ongoing but not recruiting.

#### 4.5.2. AGS-16C3F (Phases I/II)

This ADC is a fully human IgG2k monoclonal antibody that binds to the AGS-16 antigen and is conjugated to MMAF via the noncleavable maleimidocaproyl linker, and is being developed by Astellas Pharma. Details as to toxicity and utility are given in the recent papers by Donaghy [28] and Thomas et al. [44].

- NCT02639182: A Study of AGS-16C3F vs. Axitinib in Metastatic Renal Cell Carcinoma. Phase II: Actively recruiting patients.
- NCT01672755: A Study of AGS-16C3F vs. Axitinib in Metastatic Renal Cell Carcinoma Phase I: Ongoing but no further recruitment.

#### 4.5.3. Depatuxizumab Mafodotin (Phases I/II)

This ADC (also known as ABT-414) is composed of the humanized anti-EGFR antibody ABT-806 conjugated to MMAF and is in clinical trials under AbbVie. The following papers should be read by those interested in this potentially novel treatment for Glioblastoma multiforme. See Donaghy [28], Gajdosik [45], Phillips et al. [46], and Thomas et al. [29] for current information, in particular, the potential for pediatric treatment.

- NCT2573324: A Study of ABT-414 in Subjects with Newly Diagnosed Glioblastoma (GBM) with Epidermal Growth Factor Receptor (EGFR) Amplification (Intellance 1). Phase II: Study currently recruiting.
- NCI02343406: Adult Study: ABT-414 Alone or ABT-414 Plus Temozolomide vs. Lomustine or Temozolomide for Recurrent Glioblastoma Pediatric Study: Evaluation of ABT-414 in Children with High Grade Gliomas (INTELLANCE 2). Phase II: Currently recruiting patients.
- NCT02590263: Study Evaluating ABT-414 in Japanese Subjects with Malignant Glioma. Phase I: Currently recruiting.
- NCT01800695: Evaluating the Safety and Pharmacokinetics of ABT-414 for Subjects with Glioblastoma Multiforme. Phase I: Ongoing but not recruiting.
- NCT01741727: A Study of ABT-414 in Subjects with Solid Tumors. Phase I/II: Study completed.

#### 4.5.4. PF-06263507 (Phase I)

This ADC is a humanized anti-5T4 (A1) antibody linked to monomethyl auristatin F (MMAF) through a maleimidocaproyl (mc) linker, and went into a Phase I clinical trial under the auspices of Pfizer, with a paper published showing how a payload could be calculated for this agent [47], and a very recent paper by the clinical investigators suggested the levels suggested for Phase II studies [48]. However, even though it appeared to be acting as required, as the NCT record quotes “This study was terminated prematurely before treatment in Part 2 started due to a business-related decision”.

- NCT01891669: A Study of PF-06263507 in Patients with Advanced Solid Tumors Phase I.

#### 4.5.5. GSK-2857916 (Phase I)

GSK-2857916 is a humanized mAb (J6M0) conjugated to MMAF via a maleimidocaproyl linker, thus it is not cleavable. The commentary by van Rhee [49] should be read in conjunction with the report by Tai et al. [50], in order to see the potential value of this agent.

- NCT02064387: Dose Escalation Study to Investigate the Safety, Pharmacokinetics, Pharmacodynamics, Immunogenicity and Clinical Activity of GSK2857916. Phase I.

#### 4.5.6. MEDI-547 (1C1-mcMMAF)

This ADC is listed by de Goeij and Lambert [51] as being in a Phase I trial but no current records are shown in the NCT database. However, in 2014 it was reported that a Phase I trial (NCT 00796055) was discontinued due to toxicity. The details of this terminated trial are shown below. It follows that authors need to check current NIH clinical trials data, at the time of publishing, when deciding if an ADC is still viable. For the record, this ADC was generated by conjugating the fully human IgG1 anti-EphA2 monoclonal antibody (1C1) to MMAF via the stable maleimidocaproyl linker (1C1-mcMMAF).

- NCT00796055: Study of MEDI-547 to Evaluate the Safety, Tolerability, and Biologic Activity of IV Administration in Subjects with Relapsed or Refractory Solid Tumors (MEDI-54). Phase I: Discontinued (see above).

#### 4.5.7. XMT-1522 (Phase I)

This is a slightly modified MMAF (details not available as to structure) linked to a mAb targeting Her-2 and is in Phase I clinical trial under Mersana and Takeda.

- NCT02952729: Study of Antibody Drug Conjugate in Patients with Advanced Breast Cancer Expressing HER2. Phase I: This trial is currently recruiting patients.

### 4.6. Clinical Trials with Auristatin Derivatives Other Than MMAE or MMAF

#### 4.6.1. Amberstatin 269

##### ARX-788 (Phase I)

This ADC is a Her2 specific mAb conjugated to amberstatin 269 (Figure 4; **17**) that is in a trial against adult Her-2 positive metastatic breast cancer under the auspices of Zhejiang Medicine and Ambrx. Amberstatin 269 (Figure 4; **17**) is a MMAF derivative linked with a polyethylene glycol chain at the *N*-terminus.

- NCT02512237: A Dose-escalation Study of ARX788, IV Administered in Subjects with Advanced Cancers with HER2 Expression. Phase I: This trial is currently recruiting patients.

#### 4.6.2. Auristatin W

##### BAY-1129980 (Lupatumab Amadotin)

This is an ADC where the antibody is against a structural homolog of the urokinase-type plasminogen activator receptor (uPAR) and tumor-associated antigen, C4.4a, and conjugated to a cytotoxic agent based on auristatin W (*N*-demethyl-*N*-[4-(6-maleimidohexanohydrazido)-4-oxobutyl]auristatin W amide) (Figure 4; **18**). Upon intravenous administration, BAY1129980 targets and binds to C4.4a-expressing tumor cells. C4.4a, is a glycolipid-anchored membrane protein and a member of the Ly-6 family, is overexpressed by a variety of cancer cell types whereas it is minimally expressed on healthy cells. Note the structure of the agent plus linker is from a search in www.drugspider.com.

- NCT02134197: Dose-escalation Study of BAY1129980 Phase I: Currently recruiting patients.

##### BAY-1187982 (Phase I)

This ADC is an FGFR2 specific antibody linked to a derivative of auristatin W (BAY 1168650) (Figure 4; **19**). Its preclinical efficacy was described by Sommer et al. in 2016 [52]. One clinical trial was underway with studies in the USA, South Korea and Singapore with a start date of March 2015 and a completion date of July 2016, but the trial was terminated. The warhead is related to one used in BAY1129980 above.

- NCT02368951: Dose-escalation Trial of BAY1187982 in Subjects with Advanced Solid Tumors Known to Express Fibroblast Growth Factor Receptor 2 (FGFR2). Phase I: As of August 2016 the trial was terminated, with no details yet available

#### 4.6.3. Auristatin-0101

##### PF06647020 (Phase I)

This ADC is a humanized monoclonal antibody targeting tyrosine-protein kinase 7 (PTK7) conjugated to auristatin-0101 (Aur0101; Figure 4; **20**) through a valine-citrulline linker under the auspices of Pfizer and AbbVie. Details of the Pfizer synthetic efforts around the basic auristatin structure were published by Maderna et al. in an excellent review in 2014 [53].

- NCT02222922: A Study of PF-06647020 for Adult Patients with Advanced Solid Tumors. Phase I: This trial is actively recruiting patients.

## 5. Preclinical Candidates

In this section, we have described some selected molecules that have marine-derived molecules as “ADC-warheads”, but that are not yet in clinical trials, though the probabilities are that most will reach at least Phase I in the relatively near future.

### 5.1. Preclinical Auristatin Derivatives

#### 5.1.1. SGN-CD48A (Preclinical)

This is a glucuronide-linked MMAE (no structural details given) coupled to a mAb against CD48 (signaling lymphocyte activation molecule, family member 2 or SLAMF2) by Seattle Genetics [54].

#### 5.1.2. HTI-1511 (Preclinical)

This is an anti-EGFR targeted against kRAS or bRAF that has a vcPAB cleavable moiety linked to MMAE and a short (24 ethylene glycol) linker. Currently, the only report is from a recent abstract, and the construct is under the auspices of Halozyme and Abenza [55].

#### 5.1.3. mAbs 7-1C-mc-MMAF/mAbs 67-7A-mc-MMAF (Preclinical)

These are mouse mAbs raised against SAIL (Surface Antigen in Leukemia) by the California Company Ingenica Biotherapeutics linked to MMAF via the usual non-cleavable linker, with a full report in 2015 demonstrating efficacy in in vivo models in SCID mice [56].

#### 5.1.4. ASN-004 (Preclinical)

This is a humanized scFvFc antibody that targets trophoblast glycoprotein (5T4) and is conjugated to a Dolaflexin system, which contains auristatin F coupled to the carrier poly-(1-hydroxymethylethylene hydroxymethylformal). It is in development under the auspices of Mersana Therapeutics and Asana Biosciences [57].

#### 5.1.5. ZV-0201 (Preclinical)

This is an auristatin derivative with a patented linker technology used to link to an anti-Her2 mAb ZV-02. The warhead was produced by Sorrento Therapeutics [58] in the US and the antibody by Zova Bio in China. No structural details are available.

#### 5.1.6. Duostatin 3 (Preclinical)

De Goeij et al. [59] reported a modification of auristatin named duostatin 3 (Figure 5; **21**) that they linked to mAbs including tissue factor (TF), EGFR and Her-2 specific antibodies and tested them against suitable cell lines. Of their constructs, the TF-linked material was the best of the three.

### 5.2. Preclinical Candidates Not Auristatin Based

#### 5.2.1. NC-6201 (Preclinical)

This is composed of antibody–drug conjugate micelles that have the mAb NCAB001 (targeting EGFR), which is then attached to the surface of nanoparticles of self-assembled maleimide-PEG-poly-(glutamic acid benzyl ester) micelles that entrap the hemiasterlin (Figure 5; **22**) derivative E-7974 (Figure 5; **23**) covalently linked to a PEG-poly(glutamic acid benzyl ester). The activity of E-7974 was well described by Eisai scientists in 2009 [60]. E-7974 was based on the Phase II candidate HTI-286 (Figure 5; **24**) by simply replacing the *N*-terminal amino acid with pipecolic acid, which was not claimed in the original HTI-286 patent. HTI-286 (Taltobulin) was discontinued for business reasons by Wyeth prior to its takeover by Pfizer.

#### 5.2.2. MI130004 (Preclinical)

In 2013, PharmaMar scientists reported two peptides that differed only in the presence or absence of a chlorine group. The des-chloro derivative, PM-060184 (Figure 5; **25**), is currently in regular Phase II trials against cancer, whereas the choro-derivative, PM-050489 (Figure 5; **26**), is the warhead using a non-hydrolyzable linker to traztuzumab. In vitro and in vivo results showing efficacy have been reported by Pharma Mar scientists but currently only in abstract format [61,62,63].

It will be interesting to see how this construct continues as it is currently the only ADC that is using the actual toxic principle from a marine source, the sponge *Lithoplocamia lithistoides*, though due to low levels the material was then synthesized for use. All others referred to above are modifications of the original marine-derived toxins, the dolastatins or hemiasterlins.

## 6. A Potential Warhead Compound

### *DZ-2384* *(Preclinical)*

The story of the novel antitubulin marine compound diazonamide and the problems associated with determining its correct structure were well described in papers from 1991 to 2001, and the multiple total syntheses have also been well documented as can be seen in references 1 to 5 in the 2015 paper by Ding et al. describing the synthesis of DZ-2384 (Figure 5; **27**) [64].

A reason why this particular agent may well be of interest can be seen by inspection of the 2016 paper by Wieczorek et al. [65]. In this paper, the authors demonstrated the activity of this agent as a tubulin interactive agent that binds at/close to the vinca binding site, but is distinctly different in its interactions with tubulin in terms of microtubule dynamics and ultimate morphology of the tubulin molecules.

Inspection of its structure (Figure 5; **27**) shows two sites where primary alcohol groups on the molecule may be used for conjugation to suitable mAbs. That linkage to another compound does not destroy its activity can be seen from the discussion of the activities described for biotinylated DZ-2384 in the Wieczorek et al. paper [65]. Thus, even if it does not succeed as an independent drug candidate, it may well receive another lease on life as a potent warhead.

## 7. Conclusions

In this short review, we have attempted to fully update our earlier articles on this topic, and have given details on 31 ADCs that use the dolastatin derivatives originally described by the Pettit group and their collaborators. These include significant numbers based on auristatins MMAE or MMAF, together with related molecules that followed from the initial work on the MMAE/F by the Seattle Genetics group that led to the approval of brentuximab vedotin (Adcetris^®^) in 2011 by the FDA.

We have also mentioned some ADCs that are not yet in the clinic but whose in vivo activities in suitable mouse models lead us to think that they may reach Phase I clinical trial status relatively soon. These include some novel linker technologies and even the potential use of aptamer technology.

Finally, we have highlighted a very interesting derivative of the marine toxin, diazonamide, whose initial structure was wrongly assigned but whose descendent, DZ-2384, was synthesized by the Harran group (who first identified the correct structure of diazonamide). This molecule has an unusual intereaction with tubulin and it will be interesting to see what effect this interaction may have in clinical studies in man, as either the free molecule or as a warhead.

## References

[1] Newman D.J., Cragg G.M. (2014). Marine-sourced anti-cancer and cancer pain control agents in clinical and late preclinical development. Mar. Drugs.

[2] Newman D.J., Cragg G.M. (2016). Drugs and drug candidates from marine sources: An assessment of the current “state of play”. Planta Med..

[3] Pettit G.R., Kamano Y., Herald C.L., Tuinman A.A., Boettner F.E., Kizu H., Schmidt J.M., Baczynskyj L., Tomer K.B., Bontems R.J. (1987). The isolation and structure of a remarkable marine animal antineoplastic constituent: Dolastatin 10. J. Am. Chem. Soc..

[4] Pettit G.R., Herz W., Kirby G.W., Moore R.E., Steglich W., Tamm C. (1997). The dolastatins. Fortschritte der Chemie Organischer Naturstoffe; Progress in the Chemistry of Organic Natural Products.

[5] Flahive E., Srirangam J., Cragg G.M., Kingston D.G.I., Newman D.J. (2011). The dolastatins. Anticancer Agents from Natural Products.

[6] Pettit G.R., Srirangam J.K., Barkoczy J., Williams M.D., Durkin K.P., Boyd M.R., Bai R., Hamel E., Schmidt J.M., Chapuis J.C. (1995). Antineoplastic agents 337. Synthesis of dolastatin 10 structural modifications. Anticancer Drug Des..

[7] Pettit G.R., Srirangam J.K., Barkoczy J., Williams M.D., Boyd M.R., Hamel E., Pettit R.K., Hogan F., Bai R., Chapuis J.-C. (1998). Antineoplastic agents 365. Dolastatin 10 sar probes. AntiCancer Drug Des..

[8] Pettit G.R., Barkoczy J., Kantoci D. (1995). Human Cancer Inhibitory Pentapeptide Amides. EP Patent.

[9] Madden T., Tran H.T., Beck D., Huie R., Newman R.A., Pusztai L., Wright J.J., Abbruzzese J.L. (2000). Novel marine-derived anticancer agents: A phase I clinical, pharmacological, and pharmacodynamic study of dolastatin 10 (NSC 376128) in patients with advanced solid tumors. Clin. Cancer Res..

[10] Vaishampayan U., Glode M., Du W., Kraft A., Hudes G., Wright J., Hussain M. (2000). Phase II study of dolastatin-10 in patients with hormone-refractory metastatic prostate adenocarcinoma. Clin. Cancer Res..

[11] Perez E.A., Hillman D.W., Fishkin P.A., Krook J.E., Tan W.W., Kuriakose P.A., Alberts S.R., Dakhil S.R. (2005). Phase II trial of dolastatin-10 in patients with advanced breast cancer. Investig. New Drugs.

[12] Gianolio D.A., Rouleau C., Bauta W.E., Lovett D., Cantrell W.R., Recio A., Wolstenholme-Hogg P., Busch M., Pan P., Stefano J.E. (2012). Targeting her2-positive cancer with dolastatin 15 derivatives conjugated to trastuzumab, novel antibody-drug conjugates. Cancer Chemother. Pharmacol..

[13] Pettit G.R., Flahive E.J., Boyd M.R., Bai R., Hamel E., Pettit R.K., Schmidt J.M. (1998). Antineoplastic agents 360. Synthesis and cancer cell growth inhibitory studies of dolastatin 15 structural modifications. Anticancer Drug Des..

[14] Pettit G.R., Hogan F., Toms S. (2011). Antineoplastic agents. 592. Highly effective cancer cell growth inhibitory structural modifications of dolastatin 10. J. Nat. Prod..

[15] Storz U. (2015). Antibody-drug conjugates: Intellectual property considerations. mAbs.

[16] Doronina S., Senter P.D., Toki B.E. (2002). Pentapeptide Compounds and Uses Related Thereto. WO Patent.

[17] Doronina S.O., Toki B.E., Torgov M.Y., Mendelsohn B.A., Cerveny C.G., Chace D.F., DeBlanc R.L., Gearing R.P., Bovee T.D., Siegall C.B. (2003). Development of potent monoclonal antibody auristatin conjugates for cancer therapy. Nat. Biotechnol..

[18] Dorywalska M., Strop P., Melton-Witt J.A., Hasa-Moreno A., Farias S.E., Casas M.G., Delaria K., Lui V., Poulsen K., Sutton J. (2015). Site-dependent degradation of a non-cleavable auristatin-based linker-payload in rodent plasma and its effect on adc efficacy. PLoS ONE.

[19] Dorywalska M., Strop P., Melton-Witt J.A., Hasa-Moreno A., Farias S.E., Casas M.G., Delaria K., Lui V., Poulsen K., Loo C. (2015). Effect of attachment site on stability of cleavable antibody drug conjugates. Bioconj. Chem..

[20] Strop P., Liu S.-H., Dorywalska M., Delaria K., Dushin R.G., Tran T.-T., Ho W.-H., Farias S., Casas M.G., Abdiche Y. (2013). Location matters: Site of conjugation modulates stability and pharmacokinetics of antibody drug conjugates. Chem. Biol..

[21] Senter P.D., Sievers E.L. (2012). The discovery and development of brentuximab vedotin for use in relapsed hodgkin lymphoma and systemic anaplastic large cell lymphoma. Nat. Biotechnol..

[22] Flerlage J.E., Metzger M.L., Wu J., Panetta J.C. (2016). Pharmacokinetics, immunogenicity, and safety of weekly dosing of brentuximab vedotin in pediatric patients with Hodgkin lymphoma. Cancer Chemother. Pharmacol..

[23] Roth M., Barris D.M., Piperdi S., Kuo V., Everts S., Geller D., Houghton P., Kolb E.A., Hawthorne T., Gill J. (2016). Targeting glycoprotein nmb with antibody-drug conjugate, glembatumumab vedotin, for the treatment of osteosarcoma. Pediatr. Blood Cancer.

[24] Breij E.C.W., Satijn D., Verploegen S., de Goeij B.E., Schuurhuis D.H., Bleeker W.K., Houtkamp M., Parren P.W. (2013). Use of an antibody-drug conjugate targeting tissue factor to induce complete tumor regression in xenograft models with heterogeneous target expression. J. Clin. Oncol..

[25] Breij E.C.W., de Goeij B.E.C.G., Verploegen S., Schuurhuis D.H., Amirkhosravi A., Francis J., Miller V.B., Houtkamp M., Bleeker W.K., Satijn D. (2014). An antibody-drug conjugate that targets tissue factor exhibits potent therapeutic activity against a broad range of solid tumors. Cancer Res..

[26] Breij E.C.W., Verploegen S., Lingnau A., van den Brink E.N., Janmaat M., Houtkamp M., Bleeker W.K., Satijn D., Parren P. (2015). Preclinical efficacy studies using Humax-AXL-ADC, a novel antibody-drug conjugate targeting AXL-expressing solid cancers. J. Clin. Oncol..

[27] Wang X., Ma D., Olson W.C., Heston W.D. (2011). In Vitro and In Vivo responses of advanced prostate tumors to PSMA ADC, an auristatin-conjugated antibody to prostate-specific membrane antigen. Mol. Cancer Ther..

[28] Donaghy H. (2016). Effects of antibody, drug and linker on the preclinical and clinical toxicities of antibody-drug conjugates. mAbs.

[29] Thomas A., Teicher B.A., Hassan R. (2016). Antibody-drug conjugates for cancer therapy. Lancet Oncol..

[30] Infante J.R., Sandhu S.K., McNeil C.M., Kabbarah O., Li C., Zhong W., Asundi J., Wood K., Chu Y., Hamid O. (2014). A first-in-human phase I study of the safety and pharmacokinetic (PK) activity of DEDN6526A, an anti-endothelin b receptor (etbr) antibody-drug conjugate (adc), in patients with metastatic or unresectable melanoma. Cancer Res..

[31] Zhao X.Y., Subramanyam B., Sarapa N., Golfier S., Dinter H. (2016). Novel antibody therapeutics targeting mesothelin in solid tumors. Clin. Cancer Drugs.

[32] Hassan R., Thomas A., Alewine C., Le D.T., Jaffee E.M., Pastan I. (2016). Mesothelin immunotherapy for cancer: Ready for prime time?. J. Clin. Oncol..

[33] Lamberts L.E., Menke-van der Houven van Oordt C.W., ter Weele E.J., Bensch F., Smeenk M.M., Voortman J., Hoekstra O.S., Williams S.P., Fine B.M., Maslyar D. (2016). Immunopet with anti-mesothelin antibody in patients with pancreatic and ovarian cancer before anti-mesothelin antibody-drug conjugate treatment. Clin. Cancer Res..

[34] Weekes C.D., Lamberts L.E., Borad M.J., Voortman J., McWilliams R.R., Diamond J.R., de Vries E.G., Verheul H.M., Lieu C.H., Kim G.P. (2016). Phase I study of DMOT4039A, an antibody-drug conjugate targeting mesothelin, in patients with unresectable pancreatic or platinum-resistant ovarian cancer. Mol. Cancer Ther..

[35] Challita-Eid P.M., Satpayev D., Yang P., An Z., Morrison K., Shostak Y., Raitano A., Nadell R., Liu W., Lortie D.R. (2016). Enfortumab vedotin antibody-drug conjugate targeting nectin-4 is a highly potent therapeutic agent in multiple preclinical cancer models. Cancer Res..

[36] Wang J., Anderson M.G., Oleksijew A., Vaidya K.S., Boghaert E.R., Tucker L., Zhang Q., Han E.K., Palma J.P., Naumovski L. (2017). Abbv-399, a c-met antibody-drug conjugate that targets both met-amplified and c-met-overexpressing tumors, irrespective of met pathway dependence. Clin. Cancer Res.

[37] Sussman D., Smith L.M., Anderson M.E., Duniho S., Hunter J.H., Kostner H., Miyamoto J.B., Nesterova A., Westendorf L., Van Epps H.A. (2014). SGN-LIV IA: A novel antibody-drug conjugate targeting liv-1 for the treatment of metastatic breast cancer. Mol. Cancer Ther..

[38] Morrison K., Challita-Eid P.M., Raitano A., An Z., Yang P., Abad J.D., Liu W., Lortie D.R., Snyder J.T., Capo L. (2016). Development of ASG-15ME, a novel antibody-drug conjugate targeting SLITRK6, a new urothelial cancer biomarker. Mol. Cancer Ther..

[39] Pereira D.S., Guevara C.I., Jin L., Mbong N., Verlinsky A., Hsu S.J., Aviña H., Karki S., Abad J.D., Yang P. (2015). AGS67E, an anti-CD37 monomethyl auristatin E antibody-drug conjugate as a potential therapeutic for B/T-cell malignancies and AML: A new role for CD37 in AML. Mol. Cancer Ther..

[40] Mattie M., Raitano A., Morrison K., An Z., Capo L., Verlinsky A., Leavitt M., Ou J., Nadell R., Aviña H. (2016). The discovery and preclinical development of ASG-5ME, an antibody-drug conjugate targeting SLC44A4-positive epithelial tumors including pancreatic and prostate cancer. Mol. Cancer Ther..

[41] Coveler A.L., Ko A.H., Catenacci D.V., Von Hoff D., Becerra C., Whiting N.C., Yang J., Wolpin B. (2016). A phase 1 clinical trial of asg-5me, a novel drug-antibody conjugate targeting SLC44A4, in patients with advanced pancreatic and gastric cancers. Invest. New Drugs.

[42] Liu J.F., Moore K.N., Birrer M.J., Berlin S., Matulonis U.A., Infante J.R., Wolpin B., Poon K.A., Firestein R., Xu J. (2016). Phase I study of safety and pharmacokinetics of the anti-muc16 antibody-drug conjugate DMUC5754A in patients with platinum-resistant ovarian cancer or unresectable pancreatic cancer. Ann. Oncol..

[43] Boswell C.A., Mundo E.E., Zhang C., Bumbaca D., Valle N.R., Kozak K.R., Fourie A., Chuh J., Koppada N., Saad O. (2011). Impact of drug conjugation on pharmacokinetics and tissue distribution of anti-STEAP1 antibody-drug conjugates in rats. Bioconjug. Chem..

[44] Thomas L.J., Vitale L., O’Neill T., Dolnick R.Y., Wallace P.K., Minderman H., Gergel L.E., Forsberg E.M., Boyer J.M., Storey J.R. (2016). Development of a novel antibody-drug conjugate for the potential treatment of ovarian, lung, and renal cell carcinoma expressing tim-1. Mol. Cancer Ther..

[45] Gajdosik Z. (2016). Depatuxizumab mafodotin. Anti-egfr antibody-drug conjugate, treatment of glioblastoma multiforme. Drugs Future.

[46] Phillips A.C., Boghaert E.R., Vaidya K.S., Mitten M.J., Norvell S., Falls H.D., DeVries P.J., Cheng D., Meulbroek J.A., Buchanan F.G. (2016). ABT-414, an antibody-drug conjugate targeting a tumor-selective EGFR epitope. Mol. Cancer Ther..

[47] Shah D.K., King L.E., Han X., Wentland J.A., Zhang Y., Lucas J., Haddish-Berhane N., Betts A., Leal M. (2014). A priori prediction of tumor payload concentrations: Preclinical case study with an auristatin-based anti-5T4 antibody-drug conjugate. AAPS J..

[48] Shapiro G.I., Vaishampayan U.N., LoRusso P., Barton J., Hua S., Reich S.D., Shazer R., Taylor C.T., Xuan D., Borghaei H. (2017). First-in-human trial of an anti-5T4 antibody-monomethylauristatin conjugate, PF-06263507, in patients with advanced solid tumors. Investig. New Drugs.

[49] Van Rhee F. (2014). Engineering more efficacious antibody therapy for myeloma. Blood.

[50] Tai Y.-T., Mayes P.A., Acharya C., Zhong M.Y., Cea M., Cagnetta A., Craigen J., Yates J., Gliddon L., Fieles W. (2014). Novel anti-B-cell maturation antigen antibody-drug conjugate (GSK2857916) selectively induces killing of multiple myeloma. Blood.

[51] De Goeij B.E.C.G., Lambert J.M. (2016). New developments for antibody-drug conjugate-based therapeutic approaches. Curr. Opin. Immunol..

[52] Sommer A., Kopitz C., Schatz C.A., Nising C.F., Mahlert C., Lerchen H.G., Stelte-Ludwig B., Hammer S., Greven S., Schuhmacher J. (2016). Preclinical efficacy of the auristatin-based antibody-drug conjugate BAY 1187982 for the treatment of FGFR2-positive solid tumors. Cancer Res..

[53] Maderna A., Doroski M., Subramanyam C., Porte A., Leverett C.A., Vetelino B.C., Chen Z., Risley H., Parris K., Pandit J. (2014). Discovery of cytotoxic dolastatin 10 analogues with *N*-terminal modifications. J. Med. Chem..

[54] Lewis T.S., Olson D., Gordon K., Sandall S., Quick M., Finn M., Westendorf W., Linares G., Leiske C., Nesterova A. (2016). SGN-CD48A: A novel humanized anti-CD48 antibody-drug conjugate for the treatment of multiple myeloma. Blood.

[55] Huang L., Veneziale B., Frigerio M., Badescu G., Li X., Zhao Q., Bahn J., Souratha J., Osgood R., Zhao C. (2016). Preclinical evaluation of a next-generation, EGFR targeting ADC that promotes regression in *kras* or *braf* mutant tumors. Cancer Res..

[56] Kim S.Y., Theunissen J.-W., Balibalos J., Liao-Chan S., Babcock M.C., Wong T., Cairns B., Gonzalez D., van der Horst E.H., Perez M. (2015). A novel antibody-drug conjugate targeting SAIL for the treatment of hematologic malignancies. Blood Cancer J..

[57] Smith R.A., Damle N.K., Reddy S.P., Yurkovetskiy A., Bodyak N., Yin M., Gumerov D., Ter-Ovanesyan E., Qin L., Park P.U. (2015). ASN004, a novel 5T-targeted dolaflexin™ antibody drug conjugate, causes complete regression in multiple solid tumor models. Cancer Res..

[58] Miao Z., Hong Y., Zhu T., Chucholoski A.W. (2015). Drug-Conjugates, Conjugation Methods and Uses Thereof. U.S. Patent.

[59] De Goeij B.E.C.G., Satijn D., Freitag C.M., Wubbolts R., Bleeker W.K., Khasanov A., Zhu T., Chen G., Miao D., van Berkel P.H.C. (2015). High turnover of tissue factor enables efficient intracellular delivery of antibody-drug conjugates. Mol. Cancer Ther..

[60] Kuznetsov G., TenDyke K., Towle M.J., Cheng H., Liu J., Marsh J.P., Schiller S.E., Spyvee M.R., Yang H., Seletsky B.M. (2009). Tubulin-based antimitotic mechanism of e7974, a novel analogue of the marine sponge natural product hemiasterlin. Mol. Cancer Ther..

[61] Aviles P.M., Guillen M.J., Dominguez J.M., Muñoz-Alonso M.J., Garcia-Fernandez L.F., Garranzo M., Martinez V., Francesch A., Munt S., Galmarini C.M. (2014). MI130004, an antibody–drug conjugate including a novel payload of marine origin: Evidences of in vivo activity. Eur. J. Cancer.

[62] Aviles P., Guillen M.J., Dominguez J.M., Galmarini C.M., Cuevas C. (2015). MI130004, a new ADC with a payload of marine origin shows outstanding activity against her2-expressing tumors. Mol. Cancer Ther..

[63] Aviles P.M., Guillen M.J.J., Gallardo A., Cespedes M.V., Mangues R., Fiebig H., Hartman N., Dominguez J.M., Garcia L.F., Galmarini C. (2015). MI130004, a new antibody-drug conjugate, induces strong, long-lasting antitumor effect in her2 expressing breast tumor models. Cancer Res..

[64] Ding H., DeRoy P.L., Perreault C., Larivee A., Siddiqui A., Caldwell C.G., Harran S., Harran P.G. (2015). Electrolytic macrocyclizations: Scalable synthesis of a diazonamide-based drug development candidate. Angew. Chem. Int. Ed..

[65] Wieczorek M., Tcherkezian J., Bernier C., Prota A.E., Chaaban S., Rolland Y., Godbout C., Hancock M.A., Arezzo J.C., Ocal O. (2016). The synthetic diazonamide DZ-2384 has distinct effects on microtubule curvature and dynamics without neurotoxicity. Sci. Transl. Med..

